# MES Buffer Affects *Arabidopsis* Root Apex Zonation and Root Growth by Suppressing Superoxide Generation in Root Apex

**DOI:** 10.3389/fpls.2016.00079

**Published:** 2016-02-18

**Authors:** Tomoko Kagenishi, Ken Yokawa, František Baluška

**Affiliations:** ^1^Institute of Cellular and Molecular Botany, University of BonnBonn, Germany; ^2^Department of Biological Sciences, Tokyo Metropolitan UniversityTokyo, Japan

**Keywords:** root, 2-(*N*-morpholino)ethanesulfonic acid, *Arabidopsis*, ROS, transition zone, root hair

## Abstract

In plants, growth of roots and root hairs is regulated by the fine cellular control of pH and reactive oxygen species (ROS). MES, 2-(*N*-morpholino)ethanesulfonic acid as one of the Good’s buffers has broadly been used for buffering medium, and it is thought to suit for plant growth with the concentration at 0.1% (w/v) because the buffer capacity of MES ranging pH 5.5–7.0 (for *Arabidopsis*, pH 5.8). However, many reports have shown that, in nature, roots require different pH values on the surface of specific root apex zones, namely meristem, transition zone, and elongation zone. Despite the fact that roots always grow on a media containing buffer molecule, little is known about impact of MES on root growth. Here, we have checked the effects of different concentrations of MES buffer using growing roots of *Arabidopsis thaliana*. Our results show that 1% of MES significantly inhibited root growth, the number of root hairs and length of meristem, whereas 0.1% promoted root growth and root apex area (region spanning from the root tip up to the transition zone). Furthermore, superoxide generation in root apex disappeared at 1% of MES. These results suggest that MES disturbs normal root morphogenesis by changing the ROS homeostasis in root apex.

## Introduction

Good et al. (1966) selected and reported buffers with less toxicity and less reactivity to biological compounds. Since then, these buffers were introduced to enormous amount of laboratory-based experiments. Since eighties of the last century, many studies using plant hydroponic culture have been reporting the availability of MES molecule for buffering pH in liquid culture media. Imsande and Ralston (1981) demonstrated that 1–2 mM of MES solution has an excellent buffering capacity and it shows neither inhibition of nodulation nor lowering of nitrogen fixation in soybean hydroponic culture. Bugbee and Salisbury (1985) reported that MES did not significantly decrease growth as measured by the seven growth parameters in any of the five species. 5 mM of MES was shown no impact on growth or uptake of most nutrients. Potassium uptake was even enhanced by the MES buffer in non-nodulated seedlings of soybean (Schuttler, 1987).

MES, 2-(*N*-morpholino)ethanesulfonic acid, is one of broadly used Good’s buffers. This is broadly used to regulate pH value for plants culture medium, reagent solution, and physiological experiments. Since the p*Ka* value of MES is 6.15 at 20°C, it is thought to be suitable for plant growth in terms of nutrient uptake, e.g., for *Arabidopsis* at pH 5.8. Besides MES, HEPES, PIPES and MOPS are also well-known Good’s buffers as synthetic zwitterionic buffers utilizes for culture of living organisms both for animals and plants.

Meanwhile, some studies have been reporting considerable problems caused by a chemical reaction between buffer molecules and other compounds in the media. In the presence of high concentrations of MES and PIPES, the polymerization of purified tubulin was observed using electron microscope (Waxman et al., 1981). For plant experiments, MES was reported to lower nitrogen fixation and plant growth in white clover (Rys and Phung, 1985). Medeiros et al. (1993) reported that 2 mM MES in hydroponic culture reduced shoot and root dry matter yields in maize (*Zea mays* L.) and increased accumulation of N, Ca, Mg, Mn, and Zn in shoot. Miyasaka et al. (1988) reported that Mg, Mn, and Zn uptake was decreased in winter wheat. MES was reported to perturb normal growth of cucumber in hydroponic condition, because MES oxidizes Mn^2+^ to Mn^3+^ and precipitates it from the nutrient solution, and thus nutrient uptake was inhibited due to this direct chemical reaction (Stahl et al., 1999). Importantly, 4 mM MES cannot maintain pH value for long period in hydroponic condition. The pH was decreased from 6.5 to 4.0 just for 5 days (Nicholas and James, 1993). It was also reported that MES inhibits the adventitious root formation from apple stem disks at 10 mM of concentration (De Klerk et al., 2008).

In addition to compounds prepared in media such as nutrients, there are numerous biomolecules in living organisms which interact with these buffers. Reactive oxygen species (ROS) and reactive nitrogen species (RNS) are considered as important primary signaling molecules playing a role in the propagation of cellular physiological information. As an interaction with RNS, Lomonosova et al. (1998) found out the production of hydrogen peroxide in the presence of peroxynitrite (ONOO^–^; one of RNS) and HEPES buffer. HEPES was also shown to consume superoxide (one of ROS) and nitric oxide (NO) (Keynes et al., 2003). Very intriguingly, the combination of HEPES and riboflavin, often added as vitamin, in the culture medium drastically reduced NO content under the laboratory light environment (Keynes et al., 2003). Light-activated riboflavin accelerated the reaction mediated by HEPES (Keynes et al., 2003). In fact, piperazine ring-based buffers such as HEPES and PIPES have the ability to form radicals (Grady et al., 1988). These findings suggest that such buffers potentially interferes cellular ROS/RNS homeostasis. In this respect, a biochemical study demonstrated that a normal peroxidase activity for oxidizing phenolics was strongly interfered in the presence of MES at concentrations of 5 mM in the solution, due to the replacement of target substrate with MES molecule (Baker et al., 2007).

Surprisingly, 2.5–10 mM (ca. 0.05–0.2% w/v) of MES has been used as a proper concentration for culture media since *Arabidopsis* has been introduced as model plant several decades ago. It implies that plant peroxidase, one of main functions is to compose cell wall, might be affected as roots always contact to buffer-containing media. Roots require precise control of pH value and ROS homeostasis for their normal morphogenesis in different specific zones (namely; root tip, meristematic zone, transition zone, and elongation zone). Therefore, the effect of buffer, which potentially modifies the culture environments, must be assessed. Here, we observed the followings using roots of *Arabidopsis* seedlings, (1) MES enhanced the root growth as well as the number of root hairs at 0.01% (w/v) and 0.1%, whereas 1% inhibited, (2) MES enhanced the root waving phenotype, (3) MES promoted the enlargement of meristem at 0.1%, (4) 1% MES depleted apical root meristems. Less superoxide accumulation at the root apices was found in the MES-exposed roots when compared to the control roots.

## Materials and Methods

### Plant Growth Condition

Seeds of *Arabidopsis thaliana* were sterilized with 2% NaClO (ROTH, Karlsruhe, Germany) containing 0.1% Triton-X (ROTH, Karlsruhe, Germany) for 5 min. These seeds were washed in water for four times. Seeds were planted on 1/2 MS media (Duchefa, Haarlem, The Netherlands) containing 1% (w/v) sucrose (pH 5.8 with KOH) solidified with 0.4% (w/v) phytagel (Sigma, Steinheim, Germany). Each concentration of MES (Duchefa, Haarlem, The Netherlands) was added to 1/2 MS media before autoclaved. The petri dishes were incubated at 4°C for 1 day for imbibition and were put vertically at 23–25°C in 16 h light/8 h dark (light intensity: ∼120 μmol/s/m^2^, humidity: ∼50%). At the time point of 3 and 6 days, pictures of the seedlings were taken with EOS Kiss X7 (Canon, Tokyo). Root lengths were measured by ImageJ software (ver. 1.43u for Mac OSX^1^). The number of waves were counted and compared in 6 day-old roots grown in different MES concentrations.

### Microscopic Observation

Images of root hairs were taken through 0.8× objective lens of a stereomicroscope Leica MZFLIII (Solms, Germany). Images of root apices were taken through 10× objective lens of a light microscope Leica DM750 (Solms, Germany). Root hairs and distances between root tip to root growth were measured by ImageJ software.

### Histochemical NBT Staining for Superoxide Detection

To detect the presence of superoxide in root apex grown in different concentrations of MES, 6 day-old seedlings were incubated for 5 min in the staining solution of 300 μM nitroblue tetrazolium salt (NBT; Fluka, Germany) dissolved in 0.1 M Tris-HCl, 0.1 M NaCl, 0.05 M MgCl_2_ (pH = 9.5). Seedlings were then observed and imaged under a light stereomicroscope.

### Statistical Analysis

All numerical data obtained here were analyzed and tested in appropriate statistical methods. Tukey’s HSD (honestly significant difference) was applied to test a level of significance at *p* < 0.05 using R software (R for Mac OS X Cocoa^2^).

## Results

### Effect of MES Buffer on Root Growth

*Arabidopsis* Col-0 seeds were germinated and grown on the media containing four different concentrations of MES (0, 0.01, 0.1, and 1%). The MES-containing media at this range of concentration showed no effect on seed germination (data not shown). The root length was then measured at the time point of 3 and 6 days after germination.

**FIGURE 1:**
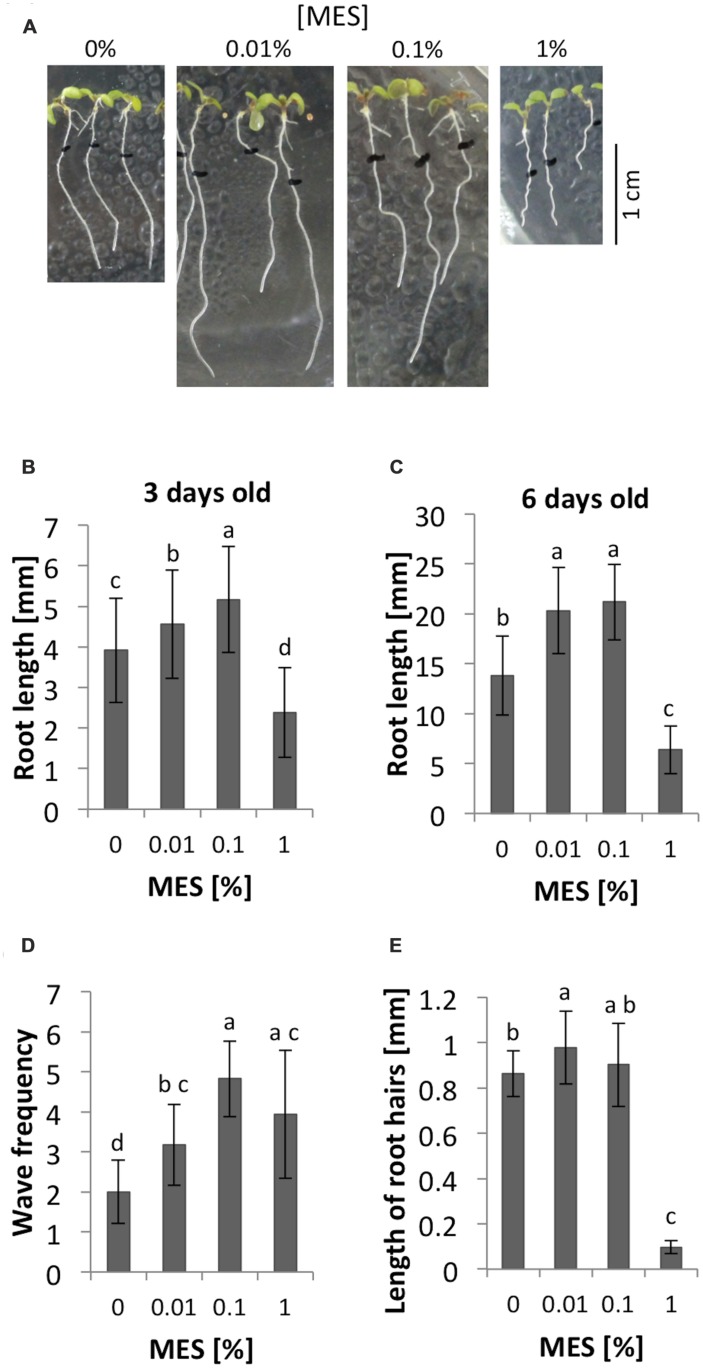
**Root growth and morphology in different concentrations of MES.** **(A)** Appearances of seedlings at 6-day old. The seeds were germinated on 1/2 MS medium containing each concentration of MES. Black marks indicate positions of root apices at 3-day old. **(B)** Root lengths at 3-day after germination on each concentration of MES. Error bars indicate standard deviation of the mean (*n* = 62–72). **(C)** Root lengths at 6-day after germination old on each concentration of MES. Error bars indicate standard deviation of the mean (*n* = 34–65). **(D)** The number of waves of the roots. Error bars indicate standard deviation of the mean (*n* = 17). **(E)** Root hair formation affected by MES. Lengths of root hairs were measured at position where long root hairs emerge, because the distance from the tip varies under different MES treatments. Five root hairs were measured from five seedlings. Error bars indicate standard deviation of the mean (*n* = 25). Different letters indicate significant difference (Tukey’s HSD test, *P* < 0.05).

Three days after germination, the average of root lengths showed significant different among 0, 0.01, 0.1, and 1% MES treatments. As **Figure 1B** shows, 0.01 and 0.1% of MES promoted the growth whereas 1% of concentration inhibited. Similarly, 6 days after germination, both 0.01 and 0.1% of MES still promoted the root growth (**Figures 1A,C**). These data indicates low MES concentration (in the range from 0.01 to 0.1%) in 1/2 MS phytagel enhances root growth from early developmental stage of seedlings, however, 1% MES inhibited. In addition to the growth, we observed the effect of MES on root growth behavior, so-called waving phenotype, which is known to reflect a root tropic growth affected by several physical factors such as gravity, light, touch etc. (Okada and Shimura, 1990; Simmons et al., 1995). Therefore, the frequency of root waving on each MES containing plate was also assessed. In the result, waving frequency was enhanced as MES concentration increased (**Figures 1A,D**).

As we described in the introduction part, pH and ROS have an important role for root growth. The formation of root hair also requires fine control of those parameters. Here the impacts of MES on root hairs in 6 days after germination seedlings were compared. Images of roots were taken through 0.8× objective lens of a stereomicroscope. As **Figure 1E** shows, 1% of MES drastically inhibited normal root hair formation. Lengths of five root hairs from five different roots (*n* = 25) in each treatment were measured and averaged. In the result, MES increased the length of root hairs when it is at 0.01% (*p* < 0.05) compared to control, but not at 0.1%. Interestingly, 1% MES strongly suppresses the formation (**Figure 1E**). The total number of root hairs did not show significant differences among four MES concentrations (data not shown).

### Root Morphology in Apex Region in the Presence of MES

**FIGURE 2:**
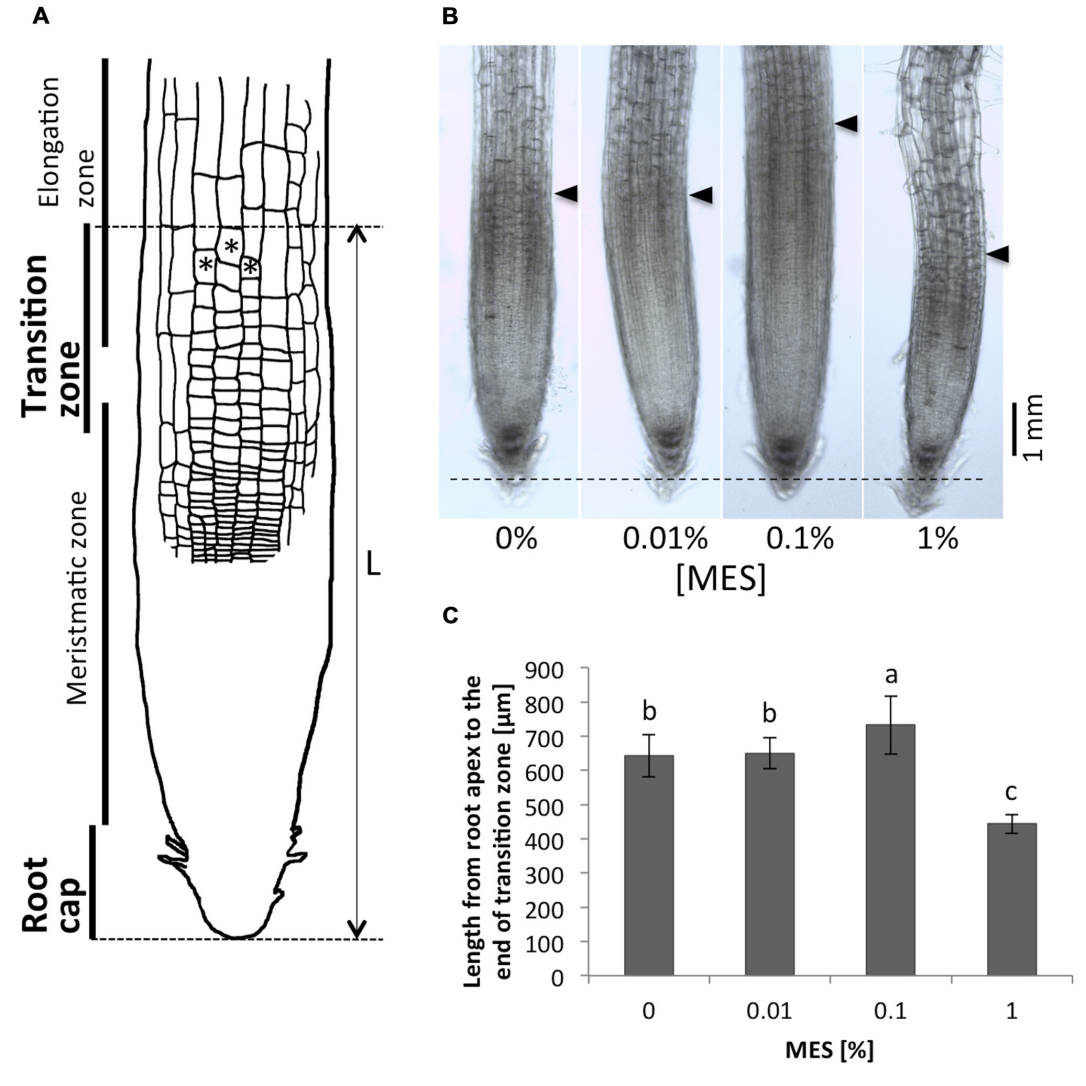
**Comparison of the root apex lengths.** **(A)** A scheme of the root apex. Cells with asterisks indicate the typical cells in transition zone. In this study, we define the border between transition zone and elongation zone as following morphological category: the cell lengths obtain values which are two times higher as the cell widths (Baluška et al., 2010). **(B)** Microscope images of the root apices. Tips of black triangulates indicate positions of the end of transition zone. A dashed horizontal line indicates the position of root apices. **(C)** Lengths of the root apices (from the root tip to the end of the transition zone). Error bars indicate standard deviations of the mean (*n* = 9–11). Different letters indicate significant difference (Tukey’s HSD test, *P* < 0.05).

**FIGURE 3:**
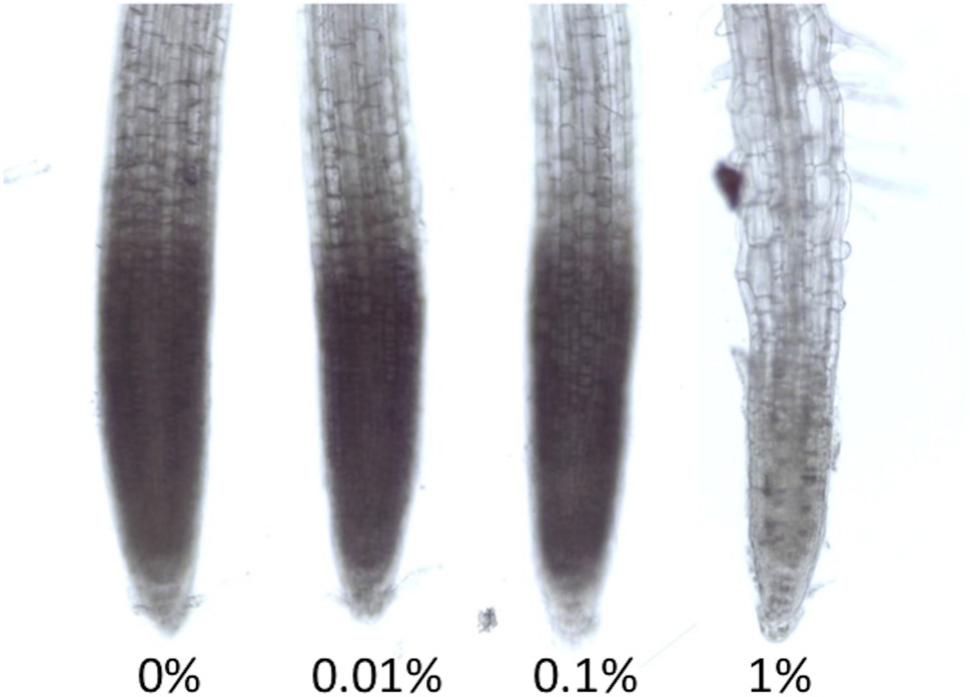
**Light Stereomicroscope pictures of NBT staining for superoxide detection.** Seedlings were grown in different concentrations of MES for 6 days. These were incubated for 5 min in NBT. The representative picture is shown here (*n* = 5).

As we observed, the presence of MES changed the root growth and its tropic behavior. Next, we have focused on root apex region in roots grown in different MES concentrations. This region is known to play an important role for polar auxin transport, which controls root tropic behaviors, and it is most sensitive part of roots to external environments. **Figure 2A** shows a scheme of root apices. Cells with asterisks indicate the typical cells in the transition zone. In this study, we have defined borders between transition zone and elongation zone as following morphological category: the cell lengths obtain values which are two times higher as the cell widths (Baluška et al., 2010). Arrows in **Figure 2B** indicate positions of the border between transition zone and elongation zone. A dashed horizontal line indicates the position of root tips. As depicted in **Figures 2B,C**, the treatment of MES resulted in altering the size of root apex region including transition zone. MES at 0.1% concentration significantly enlarged the size of area, whereas 1% minified. We observed that only the number of cells in root apex region (from the root tip to the elongation zone) was affected by MES exposure, not cell size, namely 10–15 μm of longitudinal cell length (growth direction) in the region was observed in all MES treatments. Interestingly, the abnormality in cell shape in root apex was observed in 1% MES growth condition (also shown **Figure 3**). It suggests that high MES condition might interfere with the overall root morphogenesis similarly to root hair formation (**Figure 1E**)

### Superoxide Localization in Root Apex

As we have described in the introduction part, MES was reported to interact or interfere with biological redox machineries. Many studies have already revealed that proper control of redox homeostasis or ROS signaling are ultimately essential for root growth and root tropisms. Here we tried to detect the distribution of superoxide in roots of *Arabidopsis* using a histochemical staining method, NBT staining. When NBT compound reacts with superoxide, formazan visualized as blue precipitation in cells is immediately formed. Superoxide is known as one of ROS existing in apex region, which controls cell proliferation (Tsukagoshi et al., 2010). As shown in **Figure 3**, NBT staining pattern in root apex region was observed in control, 0.01 and 0.1% MES condition. However, it completely disappeared in the 1% MES growth condition. It means that superoxide required for normal root growth in apex region is continuously diminished by 1% MES in growth media during culture on a plate for 6 days.

## Discussion

### MES Effect to pH and ROS Homeostasis

Because of the p*Ka* value in acidic region, MES compound has been used for pH buffer by adding to plant culture media in terms of nutrient uptake. In *Arabidopsis* research, MES was also introduced to buffer pH value in agar-solidified plate culture. In this study, we have demonstrated that the effect of MES on *Arabidopsis* root growth, morphogenesis and tropic behavior. Furthermore, staining superoxide in root apex indicated that MES strongly interferes with ROS homeostasis at 1% of MES, but not at 0.1%, which has an important role for root growth.

Foreman et al. (2003) demonstrated that superoxide produced by plasma membrane-associated NADPH oxidase is necessary for intracellular Ca^2+^ elevation leading to root hair formation and cell expansion. The treatment of diphenylene iodonium (DPI), inhibitor for flavo-proteins including NADPH oxidase, resulted in suppressing ROS accumulation and lacking root hairs (phenocopy of *rhd2* mutants; Foreman et al., 2003). The inhibitory effect of 1% of MES on root growth and root hair formation can be concluded as the result of disruption of superoxide production (**Figure 3**).

Besides ROS homeostasis, pH is also important factor for cell expansion and root growth regulation. It was already reported that root hair formation requires acidification of cell wall (Bibikova et al., 1998), interplay between extracellular pH and ROS production (Monshausen et al., 2007). Therefore, the interruption of root hair initiation observed here might be interpreted due to a high buffering capacity of MES at 1% concentration in the phytagel media. On the other hand, 0.01 and 0.1% MES treatment significantly enhanced root growth (**Figures 1A–C**), and 0.1% MES enhanced root hair length (**Figure 1E**) compared to control conditions. This is probably because of modest buffering ability at this range of concentrations (0.01–0.1%) support continuous acidification required for root hair tip growth as well as for the root growth.

### MES Impacts on Transition Zone and Tropism of Roots

In this study, we found out that 0.1% MES increased the area of apex region including from root tip to the border between transition zone and elongation zone (**Figure 2**). With stereomicroscope, the increase of cell numbers in this root apex region was observed. Transition zone of the root apex plays an important role for all root tropic behaviors based on polar auxin transport, which is accomplished via endocytic vesicle recycling as found in cells of this region (Baluška et al., 2010). As we have observed, the enlargement of the region in the presence of 0.1% MES (**Figure 2**) enhances waving phenotype of growing roots (**Figure 1D**). This root waving phenotype is known as a result of root tropic response of physical contact to agar surface (Okada and Shimura, 1990; Simmons et al., 1995). It was also reported that this phenotype became stronger if roots grown in light condition (Okada and Shimura, 1990). In addition, root growth pattern (waving and skewing) was also reported to change by the hardness of agar medium. 1.5% agar medium show stronger slanted-growth than 0.8% or 3% (Rutherford and Masson, 1996). Huang et al. (1995) showed that the rigidity of phytagel was altered by pH or the concentrations of nutrient components in media. Since root growth patterns depend on environmental factors (e.g., light and gravity) as well as physical contacts to media, high buffer contents are possibly changing physical properties of agar, affecting root growth on solidified plates. Very interestingly, the size of root apex correlates with the waving frequency (**Figures 1D** and **2**), suggesting that the MES-induced enlargement of this root apex region results in changing root tropic behavior.

Tsukagoshi et al. (2010) reported that UPBEAT1, a transcription factor, regulates the expression of peroxidase in root apex, determining the borderline between transition zone and elongation zone. There are two different ROS types present in these two root apex zones playing distinct roles, hydrogen peroxide in elongation zone and superoxide in transition zone is for cell differentiation and is for cell proliferation, respectively. In response to environmental conditions, roots regulate mode of growth by modulating the position of this borderline via UPBEAT1 regulation.

**FIGURE 4:**
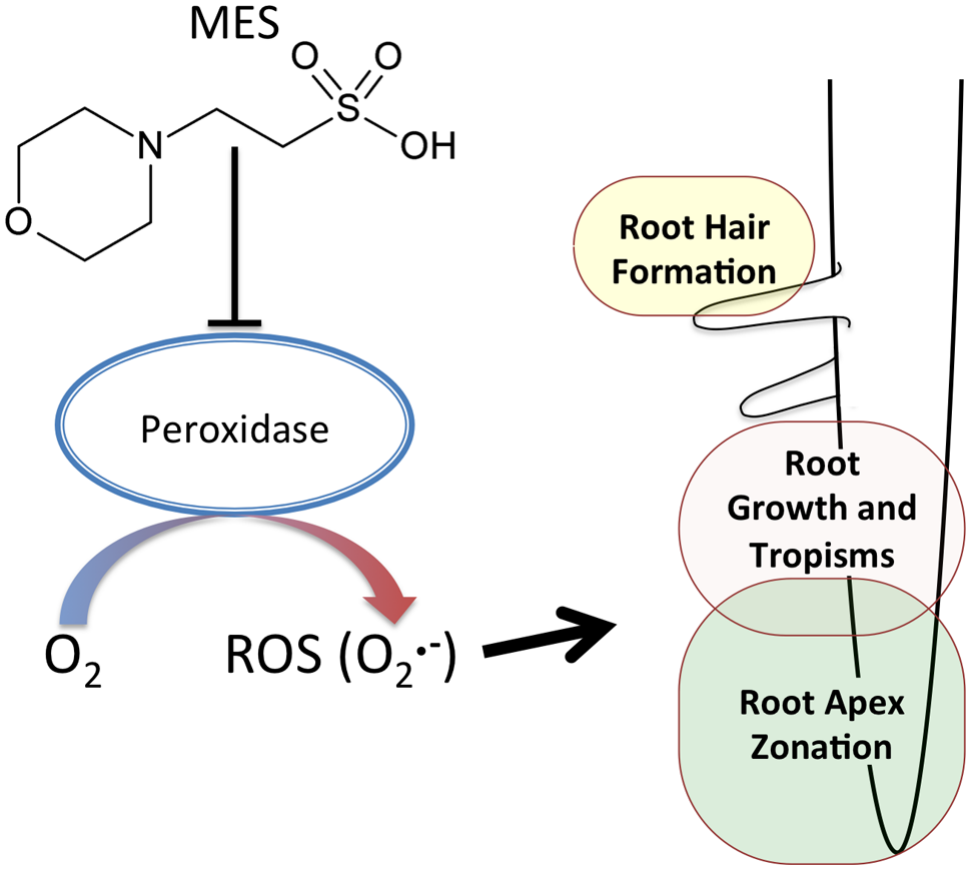
**A schematic diagram of MES effects on root growth.** MES disturbs ROS-generating pathway in the root apex, possibly via enzymes (e.g., peroxidases), affecting the root apex zonation. The ROS ($O_{2}^{•-}$) is involved in root hair formation, root growth and root tropisms.

Possible reasons for MES-altered the area of transition zone can be considered as follows. (1) Indirect effect due to MES buffering ability. Similar to ROS in roots, pH must also be controlled in different zones in roots (He et al., 2015). Therefore, roots changed the morphology as a result of an attempt to recover pH homeostasis or escape from such a situation. (2) Direct interaction of MES with peroxidases. It was already reported in a biochemical study as aforementioned. Because of the molecular structure of MES, it interferes with peroxidase activities oxidizing phenolic compounds (Baker et al., 2007). Peroxidases in root apex region might be affected in the presence of MES. It was reported that the appropriate control of ROS homeostasis by cell wall peroxidase is essential to regulate root cell elongation (Liszkay et al., 2004). As **Figure 3** shows, since superoxide in transition zone was completely disappeared at 1% concentration, it is likely that MES disturbs ROS-generating pathway, possibly via enzymes (peroxidases) or direct scavenging. A schematic summary is shown in **Figure 4.**

### MES for Laboratory-based Experiments

In addition to extracellular interaction of MES and biological compounds, it is likely that MES compound is also taken up by roots and transported to other tissues in plant body such as leaves. This suggests that incorporated MES molecule in cellular space might cause lasting reactions interfering with many extracellular and intracellular signaling molecules. For example, HEPES buffer, structurally analogous to MES, drastically consumes endogenous NO in the presence of riboflavin under laboratory light condition (Keynes et al., 2003). Culturing plants under light in a growth chamber needs great caution if growth media contain such buffers. Impacts of light on growing *Arabidopsis* roots have been reported recently. Under illuminated condition, roots alter their physiological conditions, growth rate, tropisms (Yokawa et al., 2011, 2013, 2014; Wan et al., 2012; Kagenishi et al., 2015; Mo et al., 2015; Novák et al., 2015). Importantly, roots are evolutionarily optimized to grow in darkness in nature. Light activates root photoreceptors, or probably other light-absorbing compounds in root cells. In this regard, light may even bring unexpected results through a reaction with buffer compounds inside or outside of root cells as already discussed above (Keynes et al., 2003). Moreover, as we have noted in the introduction part, 4 mM of MES (approximately 0.1%) could not maintain pH value for 5 days in liquid media. The pH was gradually decreased from 6.5 to 4.0. Potential problems with MES were pointed out previous study (Nicholas and James, 1993). All this suggests that the long-term experiments (longer than a day), that require fine pH control in liquid or solidified media, must be interpreted with a great care.

Growth conditions, buffers, other supplemental compounds such as riboflavin (vitamins) have been chosen to grow *Arabidopsis* as a model plant for laboratory-based experiments, we must take into account such artificial environments for further root research.

## Author Contributions

All authors conceived of the research. TK and KY designed the experiments, TK conducted the experiments and analyses of data. And all authors prepared and revised the manuscript.

## Conflict of Interest Statement

The authors declare that the research was conducted in the absence of any commercial or financial relationships that could be construed as a potential conflict of interest.

## Footnotes

1 http://imagej.nih.gov/ij/

2 http://www.R-project.org

## References

[B1] Baker J. Mock N. Roberts D. Deahl K. Hapeman C. Schmidt W. (2007). Interference by Mes [2-(4-morpholino) ethanesulfonic acid] and related buffers with phenolic oxidation by peroxidase. *Free Radic. Biol. Med.* 43 1322–1327. 10.1016/j.freeradbiomed.2007.07.020 17893045

[B2] Baluška F. Mancuso S. Volkmann D. Barlow P. W. (2010). Root apex transition zone: a signalling-response nexus in the root. *Trends Plant Sci.* 15 402–408. 10.1016/j.tplants.2010.04.007 20621671

[B3] Bibikova T. N. Jacob T. Dahse I. Gilroy S. (1998). Localized changes in apoplastic and cytoplasmic pH are associated with root hair development in *Arabidopsis thaliana*. *Development* 125 2925–2934. 9655814 10.1242/dev.125.15.2925

[B4] Bugbee B. G. Salisbury F. B. (1985). An evaluation of MES (2(N-Morpholino) ethanesulfonic acid) and amberlite IRC-50 as pH buffers for nutrient solution studies. *J. Plant Nutr.* 8 567–583. 10.1080/01904168509363369 11539688

[B5] De Klerk G. -J. Hanecakova J. Jásik J. (2008). Effect of medium-pH and MES on adventitious root formation from stem disks of apple. *Plant Cell Tiss. Organ. Cult.* 95 285–292. 10.1007/s11240-008-9442-5

[B6] Foreman J. Demidchik V. Bothwell J. H. Mylona P. Miedema H. Torres M. (2003). Reactive oxygen species produced by NADPH oxidase regulate plant cell growth. *Nature* 422 442–446. 10.1038/nature01485 12660786

[B7] Good N. E. Winget G. D. Winter W. Connolly T. N. Izawa S. Singh R. M. (1966). Hydrogen ion buffers for biological research. *Biochemistry* 5 467–477. 10.1021/bi00866a011 5942950

[B8] Grady J. K Chasteen N. D. Harris D. C. (1988). Radicals from “Good’s” buffers. *Anal. Biochem.* 173 111–115. 10.1016/0003-2697(88)90167-4 2847586

[B9] He Y. Wu J. Lv B. Li J. Gao Z. Xu W. (2015). Involvement of 14-3-3 protein GRF9 in root growth and response under polyethylene glycol-induced water stress. *J. Exp. Bot.* 66 2271–2281. 10.1093/jxb/erv149 25873671 PMC4986726

[B10] Huang L.-C. Kohashi C. Vangundy R. Murashige T. (1995). Effects of common components on hardness of culture media prepared with gelrite. *In Vitro Cell. Dev.* 31 84–89. 10.1007/BF02632242

[B11] Imsande J. Ralston E. J. (1981). Hydroponic growth and the nondestructive assay for dinitrogen fixation. *Plant physiol.* 68 1380–1384. 10.1104/pp.68.6.1380 16662112 PMC426107

[B12] Kagenishi T. Yokawa K. Baluška F. (2015). Dynamic regulation of endocytic vesicle recycling and PIN2 localization in *Arabidopsis* roots under varying light qualities. *Environ. Control Biol.* (in press).

[B13] Keynes R. G. Griffiths C. Garthwaite J. (2003). Superoxide-dependent consumption of nitric oxide in biological media may confound in vitro experiments. *Biochem. J.* 369 399–406. 10.1042/BJ20020933 12366375 PMC1223083

[B14] Liszkay A. van der Zalm E. Schopfer P. (2004). Production of reactive oxygen intermediates (O_2_^⋅^-, H_2_O_2_, and ^⋅^OH) by maize roots and their role in wall loosening and elongation growth. *Plant Physiol.* 136 3114–3123. 10.1104/pp.104.044784 15466236 PMC523372

[B15] Lomonosova E. E. Kirsch M. Rauen U. Groot H. (1998). The critical role of Hepes in SIN-1 cytotoxicity, peroxynitrite versus hydrogen peroxide. *Free Radic. Biol. Med.* 24 522–528. 10.1016/S0891-5849(97)00295-5 9559863

[B16] Medeiros C. Clark R. B. Ellis J. R. (1993). Effects of mes [2(n-morpholino)-ethanesulfonic acid] and ph on mineral nutrient uptake by mycorrhizal and nonmycorrhizal maize. *J. Plant Nutr.* 16 2255–2272. 10.1080/01904169309364684

[B17] Miyasaka S. C. Checkai R. T. Grunes D. L. Norvell W. A. (1988). Methods for Controlling pH in hydroponic culture of winter wheat forage. *Agron. J.* 80 213–220. 10.2134/agronj1988.00021962008000020015x

[B18] Mo M. Yokawa K. Baluška F. Wan Y. (2015). How and why do root apices sense light under the soil surface? *Front. Plant Sci*. 6:775 10.3389/fpls.2015.00775 PMC4585147 26442084

[B19] Monshausen G. B. Bibikova T. N. Messerli M. A. Shi C. Gilroy S. (2007). Oscillations in extracellular pH and reactive oxygen species modulate tip growth of *Arabidopsis* root hairs. *Proc. Natl. Acad. Sci. U.S.A.* 104 20996–21001. 10.1073/pnas.0708586104 18079291 PMC2409255

[B20] Nicholas J. C. Harper J. E. (1993). Effect of MES [2 (N-morpholino) ethanesulfonic acid] and amberlite IRC-50 resin on nutrient pH control and soybean growth. *J. Plant Nutr.* 16 895–909. 10.1080/01904169309364582

[B21] Novák J. Černý M. Pavlč J. Zemánková J. Skalák J. Plaèková L. (2015). Roles of proteome dynamics and cytokinin signaling in root to hypocotyl ratio changes induced by shading roots of *Arabidopsis seedlings*. *Plant Cell Physiol.* 56 1006–1018. 10.1093/pcp/pcv026 25700275

[B22] Okada K. Shimura Y. (1990). Reversible root tip rotation in *Arabidopsis* seedlings induced by obstacle-touching stimulus. *Science* 250 274–276. 10.1126/science.250.4978.274 17797309

[B23] Rutherford R. Masson P. H. (1996). *Arabidopsis thaliana sku* mutant seedlings show exaggerated surface- dependent alteration in root growth vector. *Plant Physiol.* 111 987–988. 10.1104/pp.111.4.987 8756492 PMC160968

[B24] Rys G. J. Phung T. (1985). Nutrient solution pH control using dipolar buffers in studies of *Trifolium repens L. nitrogen* nutrition. *J. Exp. Bot.* 36 426–431. 10.1093/jxb/36.3.426

[B25] Schuttler P. L. (1987). *Early Macronutrient Uptake, and Partitioning. in Glycine max L. Merr.* ph. D. thesis, Oregon State University, Corvallis, OR.

[B26] Simmons C. Söll D. Migliaccio F. (1995). Circumnutation and gravitropism cause root waving in *Arabidopsis thaliana*. *J. Exp. Bot.* 46 143–150. 10.1093/jxb/46.1.143

[B27] Stahl R. Grossl P. Bugbee B. (1999). Effect of 2(N-morpholino)ethanesulfonic acid (MES) on the growth and tissue composition of cucumber. *J. Plant Nutr.* 22 315–330. 10.1080/01904169909365629

[B28] Tsukagoshi H. Busch W. Benfey P. N. (2010). Transcriptional regulation of ROS controls transition from proliferation to differentiation in the root. *Cell* 143 606–616. 10.1016/j.cell.2010.10.020 21074051

[B29] Wan Y. Jasik J. Wang L. Hao H. Volkmann D. Menzel D. (2012). The signal transducer NPH3 integrates the phototropin1 photosensor with PIN2-based polar auxin transport in *Arabidopsis* root phototropism. *Plant Cell* 24 551–565. 10.1105/tpc.111.094284 22374399 PMC3315232

[B30] Waxman P. G. Campo A. A. Love M. C. Hamel E. (1981). Induction of polymerization of purified tubulin by sulfonate buffers. *Eur. J. Biochem.* 120 129–136. 10.1111/j.1432-1033.1981.tb05679.x 6273166

[B31] Yokawa K. Fasano R. Kagenishi T. Baluška F. (2014). Light as stress factor to plant roots - case of root halotropism. *Front. Plant Sci.* 5:718 10.3389/fpls.2014.00718 PMC4264407 25566292

[B32] Yokawa K. Kagenishi T. Baluška F. (2013). Root photomorphogenesis in laboratory-maintained *Arabidopsis* seedlings. *Trends Plant Sci.* 18 117–119. 10.1016/j.tplants.2013.01.002 23395309

[B33] Yokawa K. Kagenishi T. Kawano T. Mancuso S. Baluška F. (2011). Illumination of *Arabidopsis* roots induces immediate burst of ROS production. *Plant Signal. Behav.* 6 1460–1464. 10.4161/psb.6.10.18165 21957498 PMC3256371

